# Supplementary material for: Vegetation-Dependent Response to Drought in Salt Marsh Ammonia-Oxidizer Communities
Source: Microorganisms. 2019 Dec 19;8(1):9. doi: 10.3390/microorganisms8010009 (PMC7022406; doi:10.3390/microorganisms8010009)
Supplement: Supplementary file 1 [file microorganisms-08-00009-s001.pdf]

Supplemental Material

Vegetation-Dependent Response to Drought in Salt Marsh Nitrifier Communities

Jack K. Beltz, Hayley McMahon, Isis Torres-Nunez, Anne E. Bernhard
Department of Biology, Connecticut College, New London CT 06320

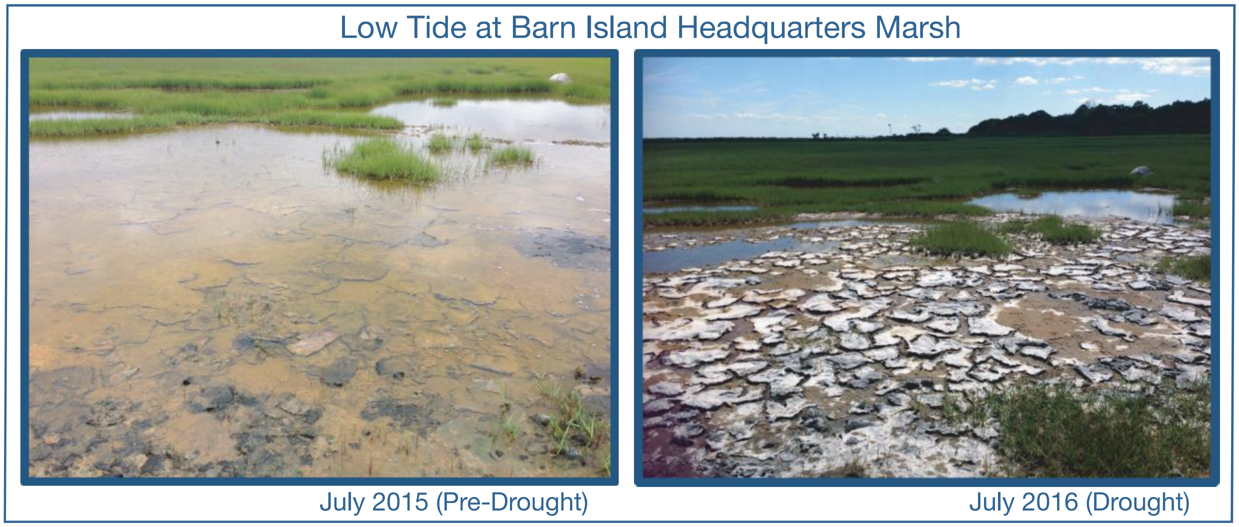

Figure S1: Images of conditions at the Headquarters Marsh of Barn Island Wildlife Management Area, Stonington CT in 2014 and 2016. Photo on the left was taken in July 2015 approximately 45 minutes after low tide (0.3 ft); photo on the right was taken in July 2016 approximately 20 minutes before low tide (0.4 ft).

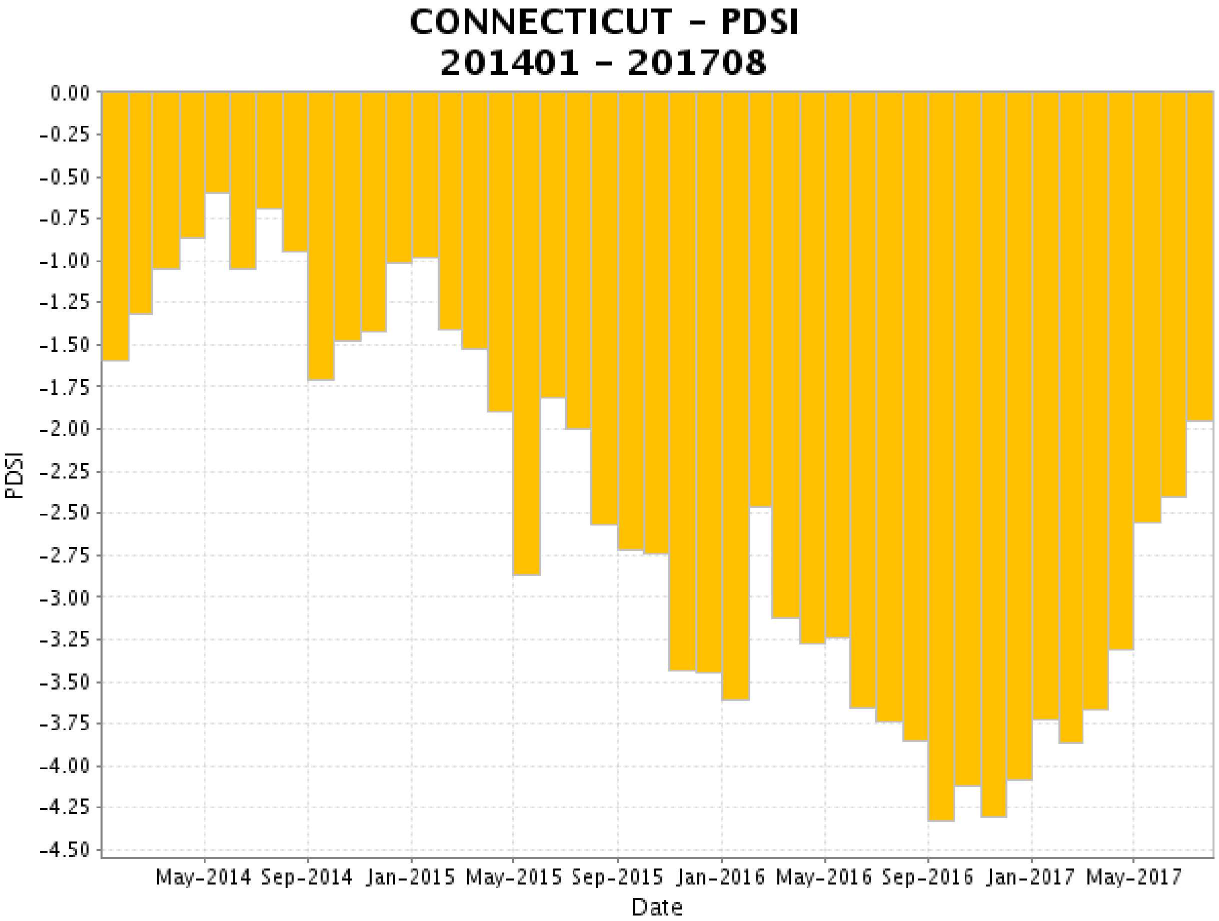

Figure S2. Palmer Drought Severity Index reported by the National Center for Environmetal Information, NOAA (https://www.ncdc.noaa.gov/cdo-web/datasets) for the study period. The data represent the conditions for coastal Connecticut. Numbers from 0.0 to -1.9 are considered near normal conditions, -2.0 to -2.9 is considered moderate drought, -3.0 to -3.9 is considered severe drought, and less than -4.0 is considered extreme drought.

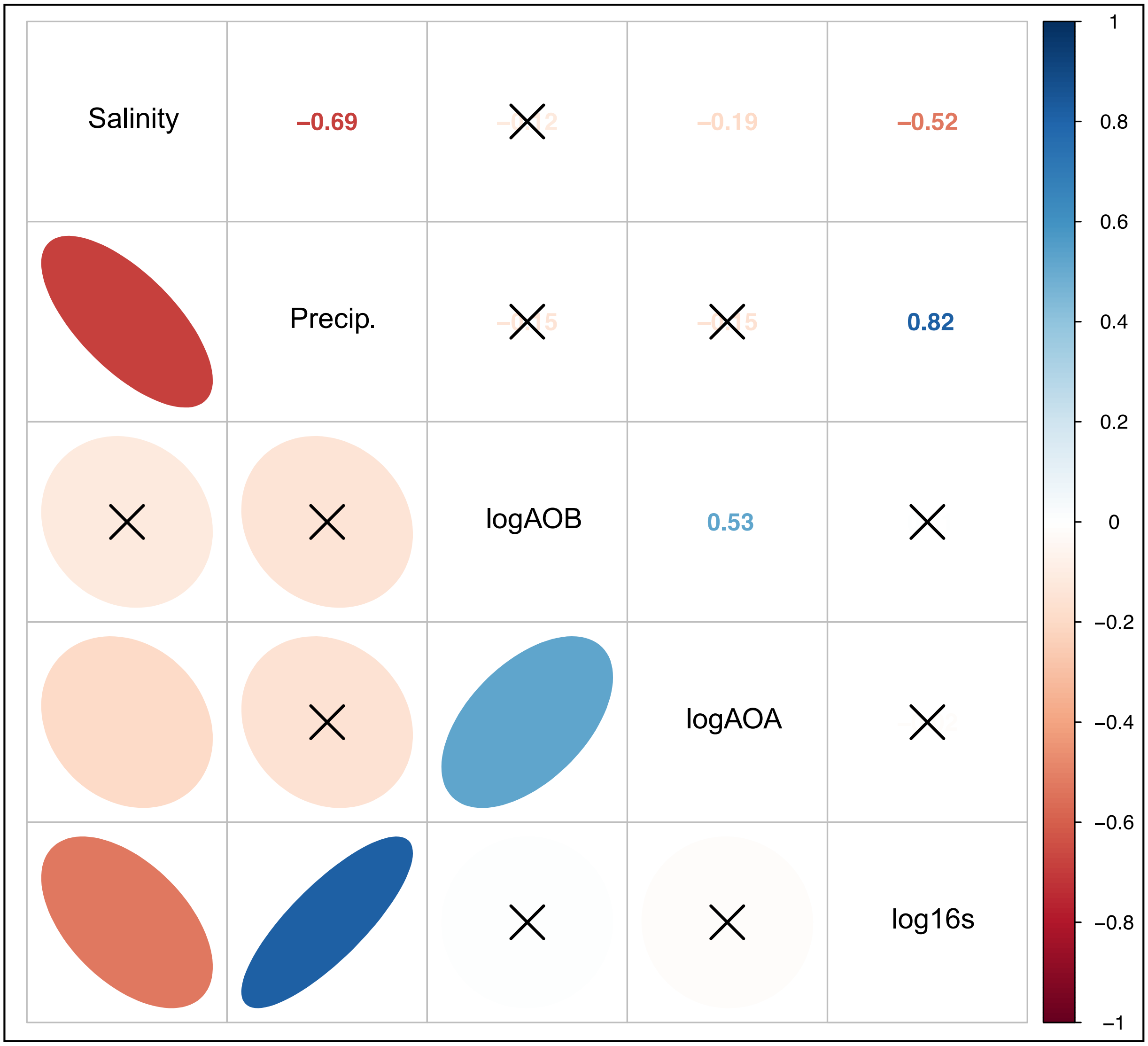

Figure S3: Correlation Plot matrix describing the relationship between salt marsh environmental variables (porewater salinity, and precipitation total one month prior to sampling) as well as the logarithmic value AOB, AOA and total bacterial abundance (copies/ gram wet sediment). Ovals show approximate shape of scatter plot, and corresponding r value placed on opposite parallel side of figure. X’s indicate non-significant relationships. Plot generated in R using “corrplot”, and “plotly”.

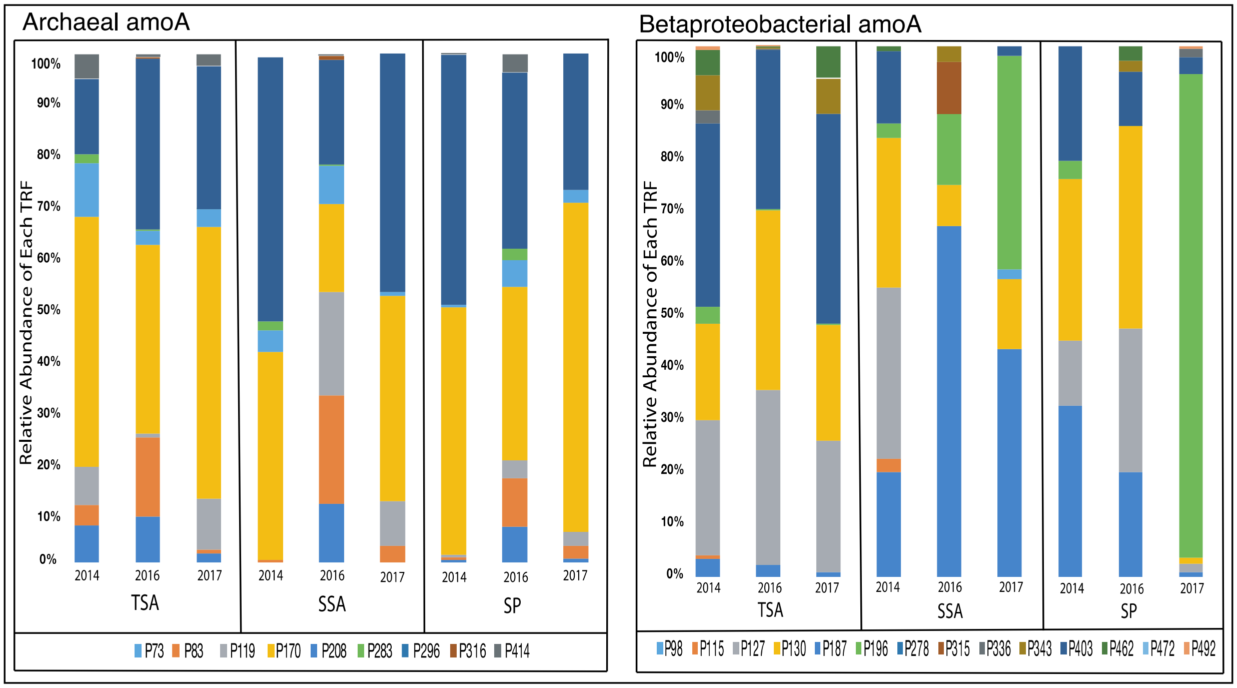

Figure S4: Terminal- Restriction Fragment Length Polymorphism community composition analysis of archaeal and betaproteobacterial ammonia oxidizers across three years and three salt marsh vegetation types. Relative abundances of unique length terminal fragments (TRF) indicated with unique colors.

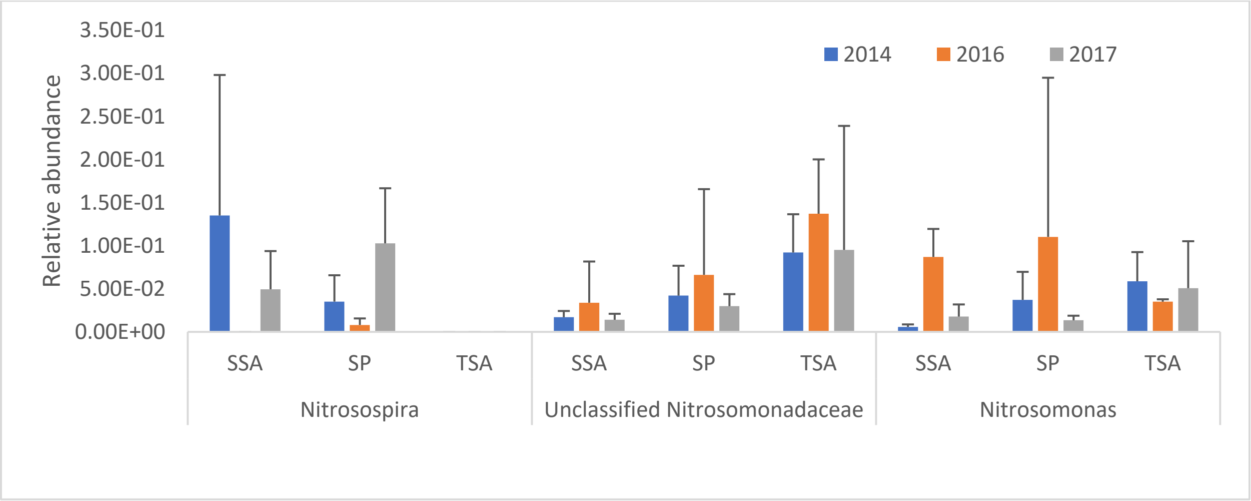

Figure S5: Average relative abundance of sequence reads per sample of three specific OTUs which were identified as members of the AOB community. Taxonomic information represents the lowest level of phylogenic classification available for those OTUs. Error bars represent the standard deviation.
